# Clinical efficacy of osimertinib for a patient with ileus due to peritoneal carcinomatosis

**DOI:** 10.1002/ccr3.2645

**Published:** 2020-01-03

**Authors:** Yo Kawaguchi, Jun Hanaoka, Hideki Hayashi, Yoshihisa Fukuda, Hirotoshi Iihara, Akio Suzuki, Tadashi Sugiyama

**Affiliations:** ^1^ Division of General Thoracic Surgery Department of Surgery Shiga University of Medical Science Otsu City Japan; ^2^ Division of General Thoracic Surgery Kusatsu General Hospital Kusatsu City Japan; ^3^ Laboratory of Pharmacy Practice and Social Science Gifu Pharmaceutical University Gifu City Japan; ^4^ Laboratory of Community Healthcare Pharmacy Gifu Pharmaceutical University Gifu City Japan; ^5^ Laboratory of Home Team Care Pharmacy Gifu Pharmaceutical University Gifu City Japan; ^6^ Department of Pharmacy Gifu University Hospital Gifu City Japan

**Keywords:** ileus, lung cancer, osimertinib, peritoneal carcinomatosis, pharmacokinetics

## Abstract

We report a patient of stage IV lung adenocarcinoma who developed ileus due to peritoneal carcinomatosis. We placed an ileus tube and started an oral intake of osimertinib. Within one month, the tumor had shrunk, and the ileus was controlled.

## Graphical abstract

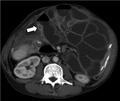

## 1 WHAT IS KNOWN AND OBJECTIVES

Peritoneal carcinomatosis arising from primary lung cancer is rare, and ileus due to peritoneal carcinomatosis is reported to be even more rare.^1^, ^2^ Despite recent medical and surgical advances, the survival of patients suffering from this condition is extremely poor, and the median survival time from diagnosis is 1.3 months.^1^ The majority of patients receive only palliative care because of their low performance status (PS).^3^

*Epidermal growth factor receptor* (*EGFR*)‐mutated lung cancer has a favorable prognosis, with a high response rate to *EGFR*‐tyrosine kinase inhibitors (TKIs); consequently, *EGFR*‐mutated lung cancer has a better overall survival time than other subtypes of lung cancer, despite the low PS of these patients.^4^ Therefore, patients with ileus due to peritoneal carcinomatosis may benefit from *EGFR*‐TKIs. The purpose of this case report is to show the clinical efficacy and pharmacokinetics of osimertinib in peritoneal metastatic lung cancer patients with ileus.

## 2 CASE DESCRIPTION

**Figure 1:**
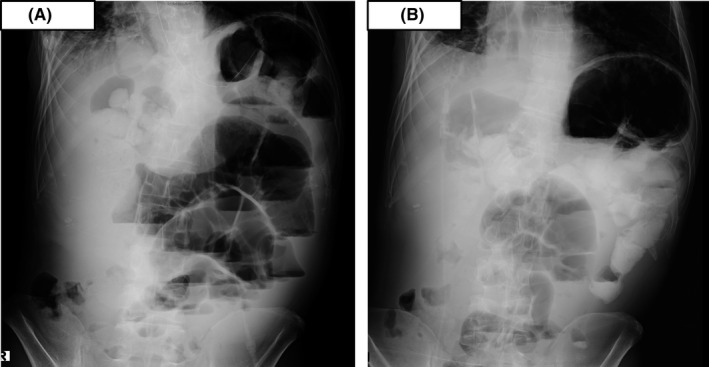
A, Abdominal X‐ray showing gaseous distension of the bowel with air‐fluid levels. B Abdominal,X‐ray showing a decrease in bowel gas and disappearance of air‐fluid levels

**Figure 2:**
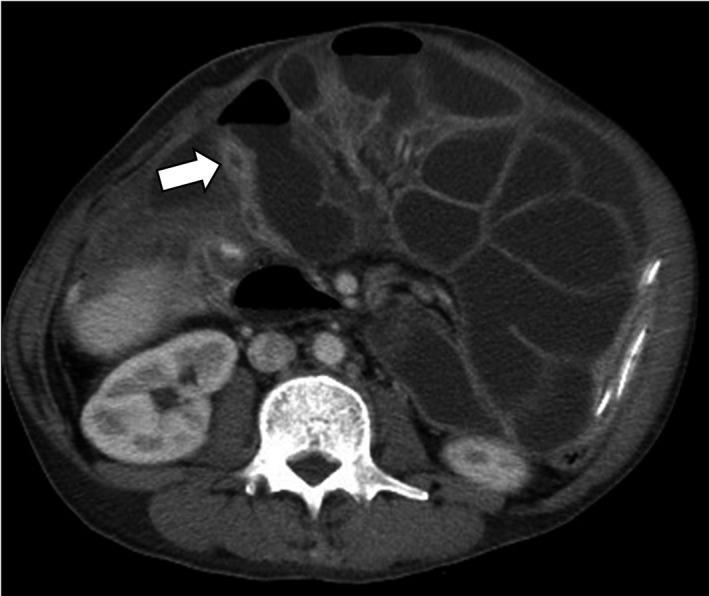
Abdominal computed tomography showing the dilatation and collapse (arrow) of the small intestine and partial thickening of the bowel wall (arrow)

**Figure 3:**
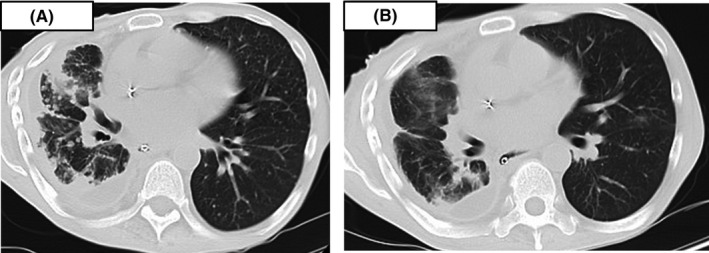
A, Chest computed tomography showing multiple lung metastases and pleural effusion. B, Chest computed tomography showing that the lung metastases disappeared

A 42‐year‐old man presented to our hospital with abnormal shadows on a chest X‐ray. He smoked 20 cigarettes per day for over 21 years. He had no history of diabetes, coronary artery disease, hypertension, hepatitis, drug allergies, or previous trauma but had previously undergone gastroduodenectomy for gastric and duodenal ulcers. Computed tomography revealed a round mass in the right upper lobe and right pleural effusion. A bronchoscopic examination was performed, and the biopsy specimen showed adenocarcinoma. The patient was finally diagnosed with stage IV lung adenocarcinoma (cT1aN0M1a) with an *EGFR exon 19 deletion* mutation. Twenty‐four treatment cycles of first‐line erlotinib 150 mg and bevacizumab 15 mg/kg led to a partial response for 17 months before multiple lung metastases developed. Additionally, four treatment cycles of second‐line carboplatin (AUC: 5) and pemetrexed (500 mg/m^2^) led to a partial response for 4 months before multiple brain metastases developed, and the patient received a whole‐brain radiation dose of 30 Gy. Three days after radiation treatment, abdominal distension, pain, and vomiting occurred. The abdominal X‐ray showed gaseous distension of the bowel with air‐fluid levels (Figure 1A), and the patient was diagnosed with ileus. Computed tomography showed ascites, dilatation, and collapse of the small intestine and a partially thickened bowel wall (Figure 2), which suggested mechanical obstruction due to peritoneal carcinomatosis. The *EGFR* gene was analyzed by real‐time polymerase chain reaction in cancer cells isolated from the peritoneal fluid and revealed adenocarcinoma with a *exon 19 deletion* and *Thr790Met* mutation. We judged that there was no indication for a surgical procedure to treat the ileus because his PS decreased to 3. Therefore, we decided to treat the patient through nonsurgical procedures. First, we placed the end of the ileus tube near the obstruction site and drained fluid and gas from the bowel for 20 days, but the ileus was not controlled. The patient was subsequently initiated on oral osimertinib 80 mg PO daily on day 1. The osimertinib had administered by mouth during the treatment period. The ileus was controlled at day 16 as evidenced by a normal bowel movement and a decrease in bowel fluid from the ileus tube facilitated subsequent removal. An abdominal X‐ray identified a decrease in bowel gas and the disappearance of air‐fluid levels (Figure 1B). Moreover, multiple lung metastases markedly reduced (Figure 3A,B). However, 4 months after starting osimertinib treatment, the patient died because of progression of the pleural carcinomatosis and multiple lung metastases.

**Figure 4:**
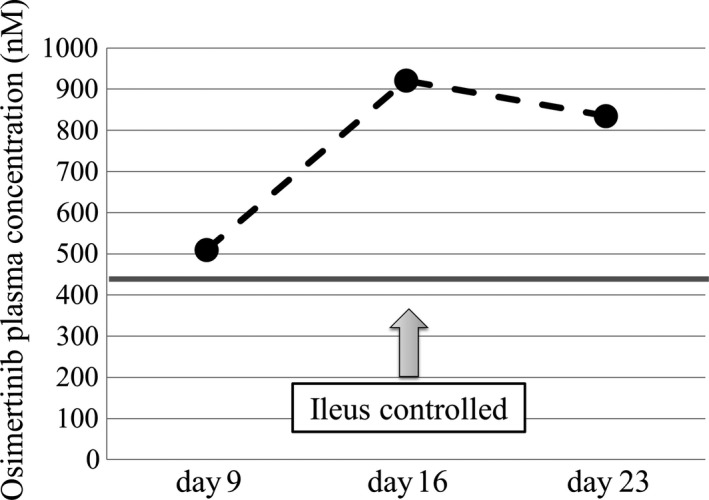
The trough plasma concentrations of osimertinib were 510 nmol/L (day 9), 921 nmol/L (day 16), and 835 nmol/L (day 23). The trough plasma concentration of osimertinib reported from an interview was approximately 440 nmol/L (bar)

Plasma samples were obtained after the administration of osimertinib at days 9, 16, and 23. The plasma concentrations of osimertinib were determined using liquid chromatography/tandem mass spectrometry according to the previously reported method^5^ with minor modifications. The trough plasma concentrations of osimertinib on days 9, 16, and 23 were 510, 921, and 835 nmol/L, respectively (Figure 4).

Before we collected samples and performed analysis, we obtained written informed consent from the patient. Institutional review board approval was also obtained for this study (approval number: 2018‐0704‐05).

## 3 WHAT IS NEW AND CONCLUSION

Peritoneal carcinomatosis arising from primary lung cancer is rare and is predominantly seen as a manifestation of intra‐abdominal malignancy, such as colorectal or ovarian cancer. Among all cases of peritoneal carcinomatosis originating from extra‐abdominal organs, 25.6% were lung cancer.^1^ Satoh et al reviewed the observations of 1041 lung cancer patients treated consecutively and found that 12 (1.2%) developed peritoneal involvement over the course of the disease.^2^ Despite recent medical and surgical advances, the survival of these patients was extremely poor, and the median survival time from the diagnosis of peritoneal carcinomatosis was 1.3 months.^1^ Peritoneal carcinomatosis sometimes causes paralytic or obstructive ileus, lending to difficulties in treatment secondary to poor patient tolerability of appropriate treatment due to poor PS.

Not only surgery, but also several treatment options are now available for patients with ileus that are not surgical candidates. Nasogastric drainage is generally a temporary measure, and self‐expanding metallic stents are an option for malignant obstructions. In terms of subsequent cancer therapy, conventional induction chemotherapy for these patients may be difficult because of their debilitated condition. Su et al reported that among 25 patients with peritoneal carcinomatosis in lung cancer, 16 patients could not receive chemotherapy due to their poor PS. Only nine patients could receive chemotherapy; however, their median survival time was 127 days.^3^ Patients without indications for conventional induction chemotherapy due to poor PS may benefit from *EGFR*‐TKIs. When *EGFR* mutation‐positive patients with PS of 3‐4 received gefitinib, the disease control rate was 90%, and 79% of the patients experienced improvements in their PS.^4^ Although oral intake is not a standard care for patients with ileus, there is likely clinical benefit of the oral administration of *EGFR*‐TKIs secondary to disease progression.

The pharmacokinetics of osimertinib in patients with ileus is unknown. We need to identify the absorption mechanisms of the drug in these patients. We believe that for effective absorption, an environment for normal bowel fluid passage needs to be created in the small intestine. First, we placed the end of the ileus tube near the obstruction site and drained fluids and gas from the bowel. Second, the patient started to orally intake osimertinib. As a result, osimertinib was absorbed through the small bowel wall, the plasma concentration of osimertinib was elevated, and the tumor markedly reduced. Tumor regression was followed by the resolution of the ileus. Kobayashi et al reported the effectiveness of afatinib in a lung cancer patient with ileus due to peritoneal carcinomatosis.^6^ The patient's ileus was also quickly controlled after afatinib treatment, which is similar to our results. A pharmacokinetic study on alvimopan, a mu opioid antagonist for postoperative ileus, demonstrated that the absorption and plasma levels of therapeutics might be higher than normal during ileus because of the longer residence time in the intestine.^7^ In the same way as this report, the plasma concentration of osimertinib during ileus was higher than that from an interview^8^ (Figure 4).

This is the first report to demonstrate the plasma concentration and marked efficacy of osimertinib in a patient with ileus due to peritoneal carcinomatosis. In conclusion, we expect that *EGFR* mutation‐positive lung cancer patients with ileus should receive *EGFR*‐TKI therapy; additionally, to adequately absorb osimertinib into the plasma, the appropriate management of oral medication intake and an ileus tube is needed.

## CONFLICT OF INTEREST

The authors have no conflicts of interest to declare.

## AUTHOR CONTRIBUTION

YK: designed the study, analyzed and interpretation of data, and wrote the manuscript. HH, YF, HI, AS, and TS: contributed to determine plasma concentrations of osimertinib using liquid chromatography/tandem mass spectrometry. JH: reviewed the manuscript. All authors approved the final version of the manuscript and agree to be accountable for all aspects of the work in ensuring that questions related to the accuracy or integrity of any part of the work are appropriately investigated and resolved.
